# Metabolomic compounds identified in *Piriformospora indica*-colonized Chinese cabbage roots delineate symbiotic functions of the interaction

**DOI:** 10.1038/s41598-017-08715-2

**Published:** 2017-08-24

**Authors:** Mo Da-Sang Hua, Rajendran Senthil Kumar, Lie-Fen Shyur, Yuan-Bin Cheng, Zhihong Tian, Ralf Oelmüller, Kai-Wun Yeh

**Affiliations:** 1 Institute of Plant Biology, National Taiwan University, 106 Taipei, Taiwan; 2 Agricultural Biotechnology Research Centre, Academia Sinica, 106 Taipei, Taiwan; 3 Institute of Natural Products, Kaohsiung Medical University, 807 Kaohsiung, Taiwan; 4 Hubei Collaborative Innovation, College of Life Science, Yangtze University, Jingzhou, 434025 Hubei China; 5 Institute of General Botany and Plant Physiology, Friedrich-Schiller-University Jena, Dornburger Str. 159, D-07743 Jena, Germany

## Abstract

Root colonization by endophytic fungus *Piriformospora indica* facilitating growth/development and stress tolerance has been demonstrated in various host plants. However, global metabolomic studies are rare. By using high-throughput gas-chromatography-based mass spectrometry, 549 metabolites of 1,126 total compounds observed were identified in colonized and uncolonized Chinese cabbage roots, and hyphae of *P. indica*. The analyses demonstrate that the host metabolomic compounds and metabolite pathways are globally reprogrammed after symbiosis with *P. indica*. Especially, γ-amino butyrate (GABA), oxylipin-family compounds, poly-saturated fatty acids, and auxin and its intermediates were highly induced and *de novo* synthesized in colonized roots. Conversely, nicotinic acid (niacin) and dimethylallylpyrophosphate were strongly decreased. *In vivo* assays with exogenously applied compounds confirmed that GABA primes plant immunity toward pathogen attack and enhances high salinity and temperature tolerance. Moreover, generation of reactive oxygen/nitrogen species stimulated by nicotinic acid is repressed by *P. indica*, and causes the feasibility of symbiotic interaction. This global metabolomic analysis and the identification of symbiosis-specific metabolites may help to understand how *P. indica* confers benefits to the host plant.

## Introduction

*Piriformospora indica* of the Sebacinales, Basidiomycota, can colonize a broad spectrum of plants^1^. The mutualistic symbiosis further promotes plant growth/biomass, and confer resistance/tolerance against biotic and abiotic stress^2, 3^. Due to its axenically cultivability, *P. indica* has been extensively used for studying the mechanisms and evolution of mutualistic symbiosis^4^. Finally, being able to promote growth significantly and deliver biotic/abiotic stress acclimation ability to its host, *P. indica* has great potential for biotechnological applications and future research for bio-agricultural engineering^5^.

The decoding of the fungal genome revealed that *P. indica* represents a missing link between a saprophytic fungus and an obligate biotrophic mutualist^6^. It holds genes for a biotrophic lifestyle, and lacks genes for nitrogen metabolism, but also matches characteristics of symbiotic fungi and obligate biotrophic pathogens^6^. The symbiotic interaction ultimately leads to growth promotion, increased biomass production, stimulation of the uptake of phosphate and other limiting ions, and enhanced tolerance to biotic and abiotic stresses, as known from mycorrhizal fungi^7, 8^. Genetic and biochemical analyses revealed possible signal transduction processes induced by *P. indica* in the root cells of host plants^9–11^. Moreover, Stein *et al*.^12^ has shown that *P. indica*-induced resistance against powdery mildew in *Arabidopsis* host involves jasmonic acid (JA), but not salicylic acid (SA). Changes in plant abscisic acid (ABA), gibberellic acid (GA), JA, and SA levels affected root colonization in barley, and changes in the GA biosynthetic pathway suppressed *P. indica*-mediated defense responses^13^. Furthermore, sustained exposure to ABA enhances the colonization potential of *P. indica* on *Arabidopsis* roots^14^. Once inside the roots, the fungus benefits from the plant’s photoassimilates, which further promotes colonization and proliferation in particular environmental stress conditions^15, 16^. As in mycorrhiza, the fungus reprograms the plant transcriptome and proteome^6, 15, 17^. The *P. indica*-responsive genes provide a powerful toolbox to elucidate signaling and metabolic pathways involved in establishing the symbiosis and promoting plant performance.

In the past decade, we studied the beneficial interaction of *P. indica* with Chinese cabbage roots^2, 7^. Besides an overall stimulation of root and shoot growth by the endophyte, the substantial increment of lateral root development results in a bushy root phenotype. Such a strong stimulation of lateral root development has not yet been described for another plant species colonized by *P. indica*. This has prompted us to investigate the symbiotic interaction of *P. indica* with Chinese cabbage roots in more details, since the strong effect of the fungus on root growth and proliferation might be important for agricultural applications. We have demonstrated that the observed phenotype is associated with a strong, transient increase of the auxin level in the roots, which results in upregulation of Chinese cabbage genes correlated with growth- and auxin signaling-related functions, such as genes for P-type/V-type H^+^-ATPases and nutrient/ion transporters, root hair-forming phosphoinositide phosphatase 4 (RHD4), cell wall loosening/synthesizing proteins involved in cell wall growth, and auxin transportation/responses^2^. Some of the genes induced during the biotrophic phase of *P. indica* colonization harmonize the auxin level with the degree of root colonization. In-depth microscopic analyses of the root structure after successful symbiosis showed that the fungus stimulates primarily growth and development of the root maturation zone^7^.

While relatively much is known about the transcriptomic and proteomic changes in roots in response to colonization by beneficial fungi, systematic analyses of metabolomic profiles are still rare. Thus far, the induction of salicylic acid catabolites and jasmonate as well as glucosinate metabolism have been demonstrated correlating with the colonization of *A. thaliana* with *P. indica* ^18, 19^. Recently, Strehmel *et al*.^20^ investigated the metabolic response of *A. thaliana* to *P. indica* under hydroponic condition. Results showed that the profiles of primary and secondary metabolites were obviously changed in colonized roots, while far less significant change in leaves. The increased concentrations were found in organic acids, carbohydrate, ascorbate, glucosinates and hydroxycinnamic acids, and a decreased concentration found in nitrogen-rich amino acids. This important finding has contributed to validate the symbiotic mechanism between plant host and *P. indica*.

Following our previous study, in order to further understand the mechanisms of stress tolerance and growth promotion in Chinese cabbage caused by colonization of *P. indica*, we performed a comprehensive metabolomic analysis, in which the profiles of *P. indica*-colonized and uncolonized Chinese cabbage roots, and the profile of *P. indica* hyphae were comparatively surveyed. The data allow the identification of symbiosis-specific metabolites in *P. indica*-colonized Chinese cabbage roots, such as GABA and IAA derivatives. The results are helpful for validating the beneficial function triggered by *P. indica-*colonization. Most importantly, the mechanisms that growth promotion and abiotic/biotic stress tolerance are simultaneously enhanced by *P. indica* could be demonstrated based on the metabolomic data.

## Results

### Metabolome profiles of *P. indica*-colonized and uncolonized Chinese cabbage roots and *P. indica* hyphae

**Figure 1:**
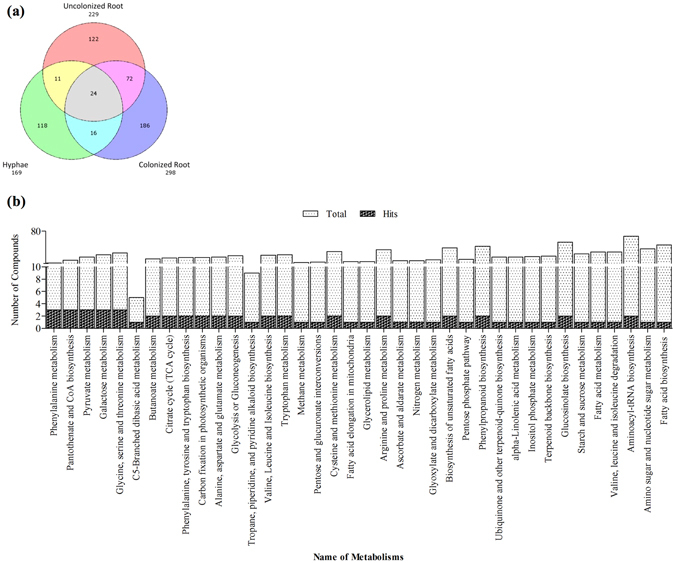
Analysis of metabolomic data from *B. campestris* roots after *P. indica* colonization. (**a**) Venn Diagram showing the number of compounds in extracts from *P. indica-*colonized roots (Pi-Root), un-colonized roots (Root Only), and hyphae of the fungus (Pi Only). (**b**) Number of identified metabolites classified according to biological functions.

In order to identify metabolites induced or repressed in Chinese cabbage roots in response to *P. indica* colonization, three metabolomic libraries were generated: two from Chinese cabbage root extracts with and without *P. indica* inoculation, and one from a hyphal extract of *P. indica*. In all, 549 of a grand total 1,126 metabolites were identified from the GC-MS analysis. Of the 549 metabolites annotation uncovered, 298 compounds in preparations from *P. indica*-colonized roots, 229 compounds from uncolonized roots and 169 compounds from *P. indica* hyphae (Fig. 1a). The compounds identified in the preparation from the *P. indica* hyphae were subtracted from those identified in *P. indica*-colonized and uncolonized Chinese cabbage roots. The compounds which were identified in the *P. indica*-colonized, but not in uncolonized roots should be induced in the symbiotic interaction, and can be of plant or fungal origin. The compounds, which were present in uncolonized roots but not detectable in *P. indica*-colonized roots should be repressed by the fungus in the roots, and were of plant origin.

Metabolites from *P. indica*-colonized root extract were characterized and classified into global metabolic pathways by using MetPA software analysis. As shown in Fig. 1b, they generally belong to the carbohydrate and amino acid metabolisms and biosynthetic pathways for secondary metabolites. This suggested that root colonization of *P. indica* reprograms the global metabolism in cabbage.

### *P. indica* induced metabolites associated with the host’s TCA cycle

**Figure 2:**
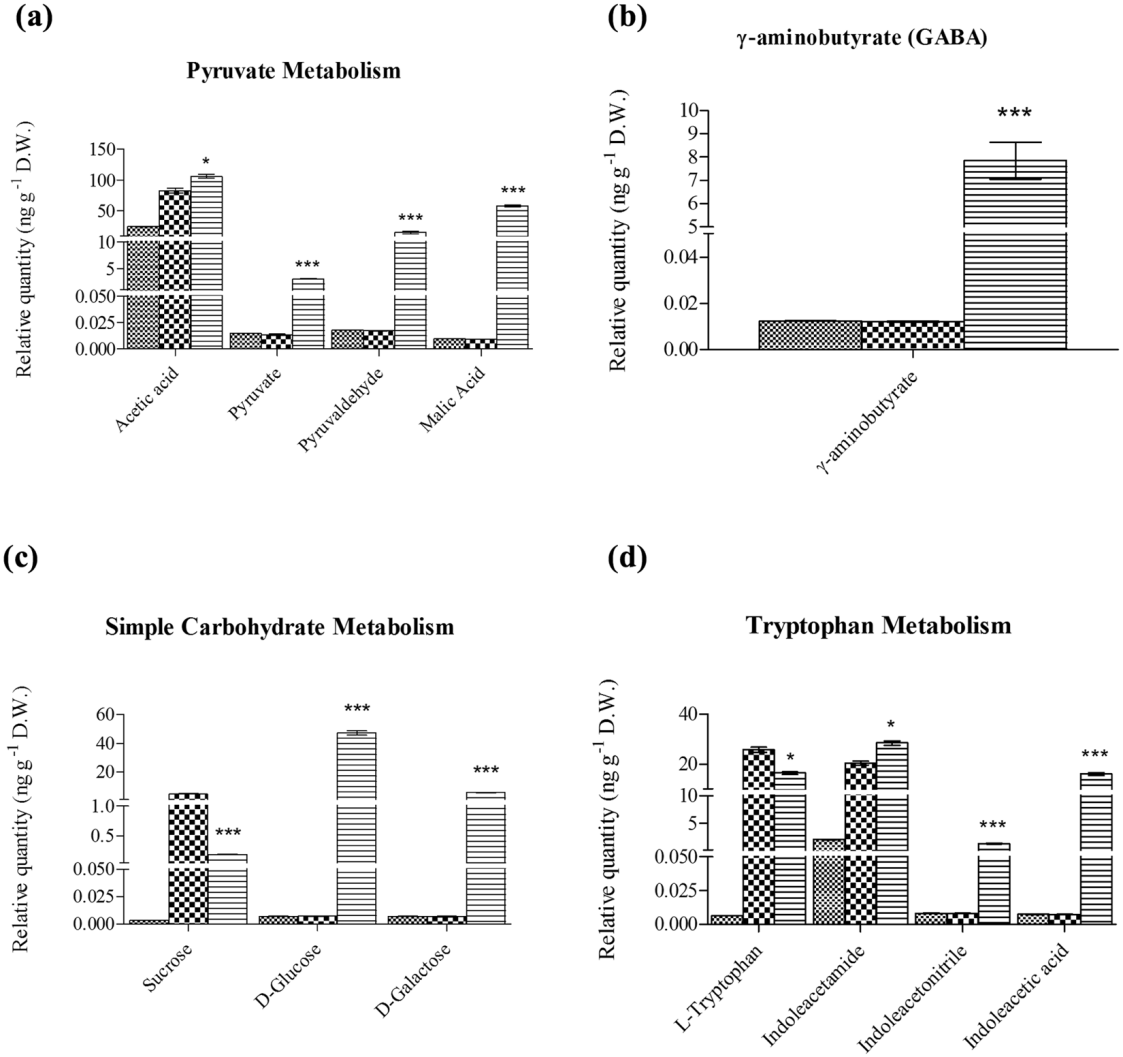
Quantification of metabolites induced by *P. indica* in *B. campestris* roots. Relative quantification of (**a**) metabolites involved in TCA cycle; (**b**) γ-aminobutyrate; (**c**) metabolites involved in simple carbohydrate metabolisms; and (**d**) metabolites from auxin biosynthesis. Standard Error (SE) was calculated by three biological repeats taken by the average of each relative quantification from peak area acquired from GC-MS data analysis, p-value < 0.01 for significant among variables *Hyphae*, *Uncolonized Root*, *Colonized Root* for each compound. Legend:  Hyphae;  Uncolonized Root and  Colonized Root.

The levels of pyruvate, malic acid as well as pyruvaldehyde relative to the internal control (nonadecanoic acid-trimethylsilyl ester) were three to five times higher (mass per gram of dry weight) for *P. indica*-colonized Chinese cabbage roots compared to the uncolonized control (Supplementary Table 1 and Fig. 2a). Notably, accumulation of pyruvaldehyde suggested that the side-products of the activated TCA cycle could not be removed fast enough in *P. indica*-colonized roots. Moreover, our previous transcriptome analyses identified mRNAs for enzymes participating in the pyruvate metabolism, which were induced by *P. indica* in Chinese cabbage roots^7^ (Table 1), such as hydroxyacylglutathione hydrolase, malate synthase and malate dehydrogenase. Therefore, *P. indica* appears to stimulate the TCA cycle activity by accumulating metabolites involved in the pyruvate metabolism as well as by inducing genes required for the production of these metabolites. Equally important, mitochondria of plant host are likely the targets of *P. indica* in Chinese cabbage roots. Finally, the results also revealed that TCA cycle activity in the colonized roots might be stimulated to generate simple carbohydrates such as glucose and galactose (Fig. 2c) through the elevated pyruvate levels.

**Table 1:** List of genes identified by subtractive EST-analysis between: *Hyphae*, *Colonized* and *Uncolonized roots*.

ORF name	Sequence length	SwissProt			
		Best hits and annotation	E-value	identity	Species
**Fatty Acid Metabolism**					
A181.seq	1112	gi|3334184|sp|Q39287.1|FAD6E_BRAJU Omega-6 fatty acid desaturase	1.00E-68	94.26	*Brassica juncea*
B93.seq	1160	gi|122223793|sp|Q0WRB0.1|FACR5_ARATH Fatty acyl-CoA reductase	1.00E-145	85.71	*Arabidopsis thaliana*
C131.seq	1146	gi|114149941|sp|Q570B4.2|KCS10_ARATH 3-ketoacyl-CoA synthase	3.00E-55	97.3	*Arabidopsis thaliana*
D176.seq	1092	gi|75166464|sp|Q94KI8.1|TPC1_ARATH Two pore calcium channel protein 1 (TPC1)	7.00E-70	94	*Arabidopsis thaliana*
H88.seq	1174	gi|3334184|sp|Q39287.1|FAD6E_BRAJU Omega-6 fatty acid desaturase	5.00E-106	94.15	*Brassica juncea*
I21.seq	1291	gi|134943|sp|P29108.1|STAD_BRANA Acyl-[acyl-carrier-protein] desaturase	3.00E-111	99.52	*Brassica napus*
**Pheylalanine metabolism**					
F92.seq	1222	gi|76803811|sp|P35510.3|PAL1_ARATH Phenylalanine ammonia-lyase 1	1.00E-53	87.2	*Arabidopsis thaliana*
C177.seq	1120	gi|21542386|sp|P46645.2|AAT2_ARATH Aspartate aminotransferase	6.00E-32	90.28	*Arabidopsis thaliana*
G71.seq	1143	gi|3915085|sp|P92994.1|TCMO_ARATH Trans-cinnamate 4-monooxygenase	2.00E-78	98.18	*Arabidopsis thaliana*
**Pyruvate Metabolism**					
F59.seq	1102	gi|11133446|sp|P57106.1|MDHC2_ARATH Malate dehydrogenase	2.00E-22	72.53	*Arabidopsis thaliana*
I2.seq	1252	gi|11133398|sp|O82399.1|MDHG2_ARATH Probable malate dehydrogenase	1.00E-158	93.14	*Arabidopsis thaliana*
D67.seq	1192	gi|3913733|sp|O24496.2|GLO2C_ARATH Hydroxyacylglutathione hydrolase	2.00E-67	92.13	*Arabidopsis thaliana*
C44.seq	1097	gi|75311439|sp|Q9LPR4.1|LEU11_ARATH 2-isopropylmalate synthase 1	3.00E-32	82.35	*Arabidopsis thaliana*
**Simple Carbohydrate Metabolism**					
J36.seq	1094	gi|60389815|sp|Q9FXT4.1|AGAL_ORYSJ Alpha-galactosidase	8.00E-36	58.02	*Oryza sativa Japonica*
B146.seq	1206	gi|122215404|sp|Q3ECS3.1|BGL35_ARATH Myrosinase 5	3.00E-05	33.66	*Arabidopsis thaliana*
F7.seq	1227	gi|75171106|sp|Q9FJU9.1|E1313_ARATH Glucan endo-1,3-beta-glucosidase 13	3.00E-40	38.1	*Arabidopsis thaliana*

List of genes identified by subtractive EST-analysis^7^ that were matched with the metabolome analysis of 7 days *P. inidica* colonized Chinese cabbage root.

### *P. indica* stimulated the synthesis of γ-aminobutyrate (GABA) and metabolites involved in the tryptophan and phenylalanine metabolism

GABA is a non-proteinous amino acid compound and accumulates rapidly in response to biotic and abiotic stress in plants^21^. It has been proposed to function as an osmo- and redox regulator and a plant-signaling molecule^21–23^. From the quantitative analysis, the GABA level was much higher in *P. indica*-colonized roots compared to the uncolonized controls, suggesting an important role in symbiosis (Supplementary Table 1 and Fig. 2b).

From our previous reports, auxin level was evidently elevated in cabbage root tissues after colonization by *P. indica*. The higher the auxin level synthesized *de novo* in *P. indica*-colonized root tissues, the more it promotes growth and in particular the development of the bushy root architecture^2, 7^. The metabolic profile showed that three intermediates of the tryptophan metabolism are prominently induced in *P. indica*-colonized roots (Supplementary Table 1 and Fig. 2d). IAM (indoleacetamide), which is already present in *P. indica* hyphae and uncolonized roots, was strongly elevated during root colonization (Supplementary Table 2 and Fig. 2d). In addition, indoleacetonitrile (IAN) and indoleacetic acid (IAA) could only be detected after root colonization (Supplementary Table 2 and Fig. 2d). In contrast, tryptophan was less abundant in the colonized roots, possibly due to the intensive biosynthetic efflux into the auxin biosynthesis pathway. Overall, the current metabolite levels corresponded to the gene expression patterns from the subtractive EST library of *P. indica*-colonized and uncolonized roots^2, 7^. The present data further supported the important role of the tryptophan and indole metabolism for *de novo* biosynthesis of auxin in *P. indica*-colonized roots, which facilitates growth promotion in Chinese cabbage^2, 7^.

**Figure 3:**
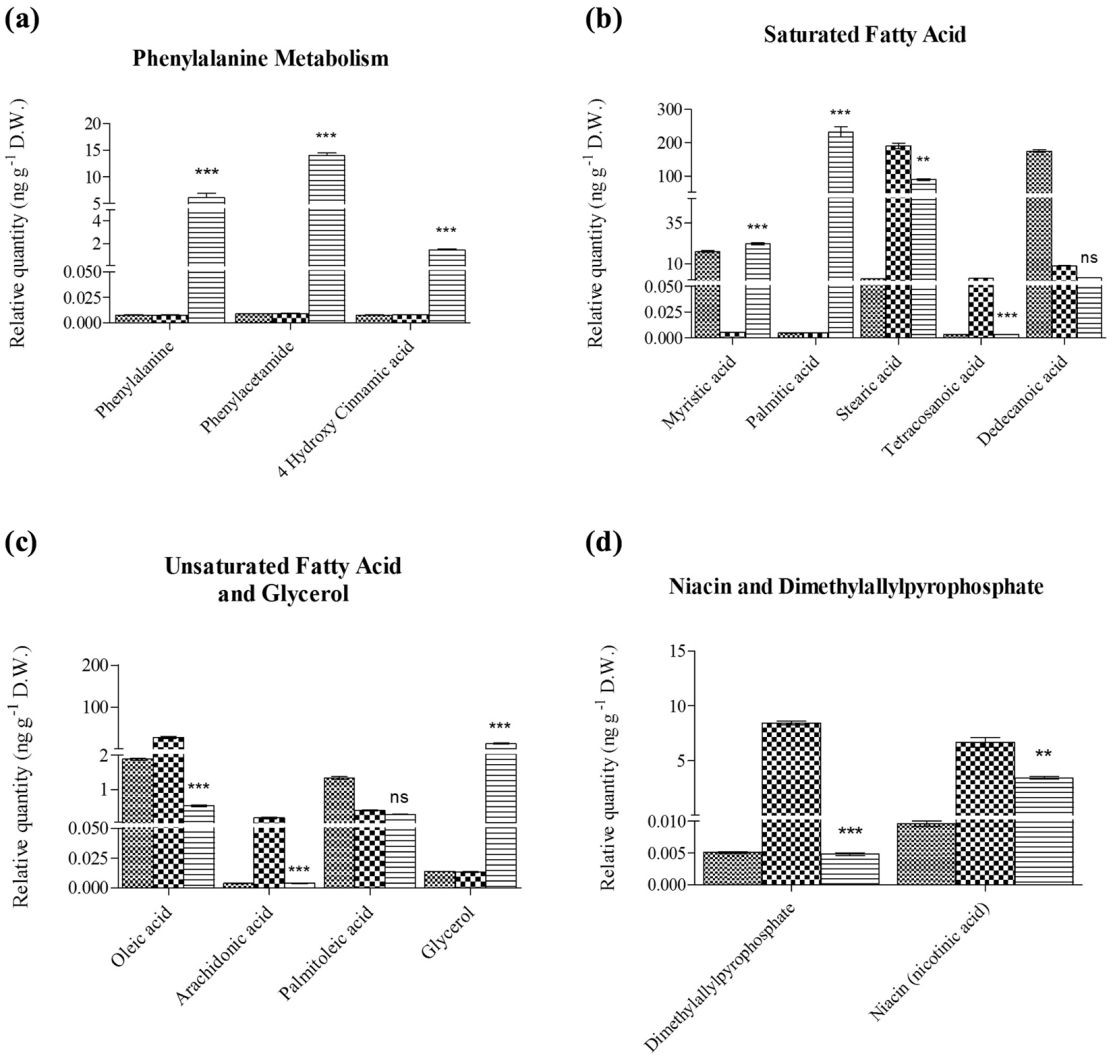
Quantification of metabolites that were reduced and altered by *P. indica* after colonization with *B. campestris* root. Relative quantification of (**a**) metabolites involved in phenylalanine metabolism; (**b**) saturated fatty acids; (**c**) unsaturated fatty acids and glycerol; and (**d**) nicotinic acid and dimethylalyl-diphosphate. Standard Error (SE) was calculated by three biological repeats taken by the average of each relative quantification from peak area acquired from GC-MS data analysis, p-value < 0.01 for significant among variables *Hyphae*, *Uncolonized Root*, *Colonized Root* for each compound. Legend:  Hyphae;  Uncolonized Root and  Colonized Root.

The tryptophan level was reduced in colonized roots, as compared to that in uncolonized roots, while auxin-biosynthetic intermediates were increased (Fig. 2d). This suggested that high amounts of tryptophan were utilized for the biosynthesis of intermediates of downstream steps in the related biosynthetic pathways. On the other hand, the compounds of the phenylalanine metabolism, such as phenylalanine, phenylacetamide and 4-hydroxy cinnamic acid were enhanced in *P. indica*-colonized roots (Fig. 3a). Coincidently, more ESTs for phenylammonia-lyase were present in the EST library of *P. indica*-colonized roots compared to a library from the uncolonized control^2, 7^ (and also in Supplementary Fig. S1). This suggested that many secondary metabolites involved in defense function were increased by *P. indica*-colonization.

### *P. indica* altered fatty acid composition and saturation in Chinese cabbage

We also observed an increase in the saturated fatty acid levels, such as myristic and palmitic acid in colonized roots (Fig. 3b). Their amounts (ng per gram dry weight) were ten times higher in colonized roots compared to uncolonized roots (Fig. 3b). In contrast, the amount of polyunsaturated fatty acids such as arachidonic acid, monounsaturated fatty acids and oleic acid decreased (Fig. 3c). Also, the glycerol level was higher (Fig. 3c) suggesting a stimulatory effect of *P. indica* on the anabolism of fatty acid biosynthesis. The increase in saturated and the decrease of unsaturated fatty acids^24^, suggested that *P. indica* primes Chinese cabbage roots to adapt to upcoming stress situations.

It has been well known that plants produce various types of oxygenated fatty acids, termed “oxylipins”, in responses to microbial infection or biotic/abiotic stresses. Usually, α-linolenic acid and arachidonic acid (C18:2 and C18:3) were the major precursors oxidized to 2-hydroperoxy by lipoxygenase^25, 26^. Jasmonic acid (JA) was present in *P. indica*-colonized roots, but below detectability in uncolonized roots and *P. indica* hyphae (Supplementary Table 1). Moreover, only low levels of the oxylipin precursors arachidonic acid (Fig. 3c) and linolenic acid (Supplementary Table 2, marked by*) were found in colonized roots relative to the uncolonized control. This demonstrated that the oxylipin production in the host was stimulated by *P. indica*, and that the endophyte was capable to induce oxygenated fatty acids (oxylipins) to promote stress tolerance.

### Niacin and dimethylallyl pyrophosphate (DMAP) were repressed by *P. indica* in Chinese cabbage roots

*P. indica* reduced the niacin level in Chinese cabbage roots to half the amount of uncolonized roots (Fig. 3d). Niacin and other vitamin B complexes has been known to elevate plant innate immunity against fungal attack^27^. Thus, reducing the niacin level might reduce innate immunity and promote *P. indica* colonization. Likewise, dimethylallylpyrophosphate, an intermediate of the zeatin and ubiquinone biosynthesis, was reduced by *P. indica* (Fig. 3d). Zeatin was required for the synthesis of the terpenoid backbone, e.g. for cytokinins and sterols. Consequently, the levels of stigmast-5-en-3-ol and squalene seemed reduced after *P. indica* colonization (Supplementary Fig. S2b). This resulted in lower cytokinin levels, and an increase in the auxin to cytokinin ratio favorable for promoting root development.

### Exogenous application of the GABA homolog, BABA, and niacin on Chinese improved abiotic and biotic stress adaptation

**Figure 4:**
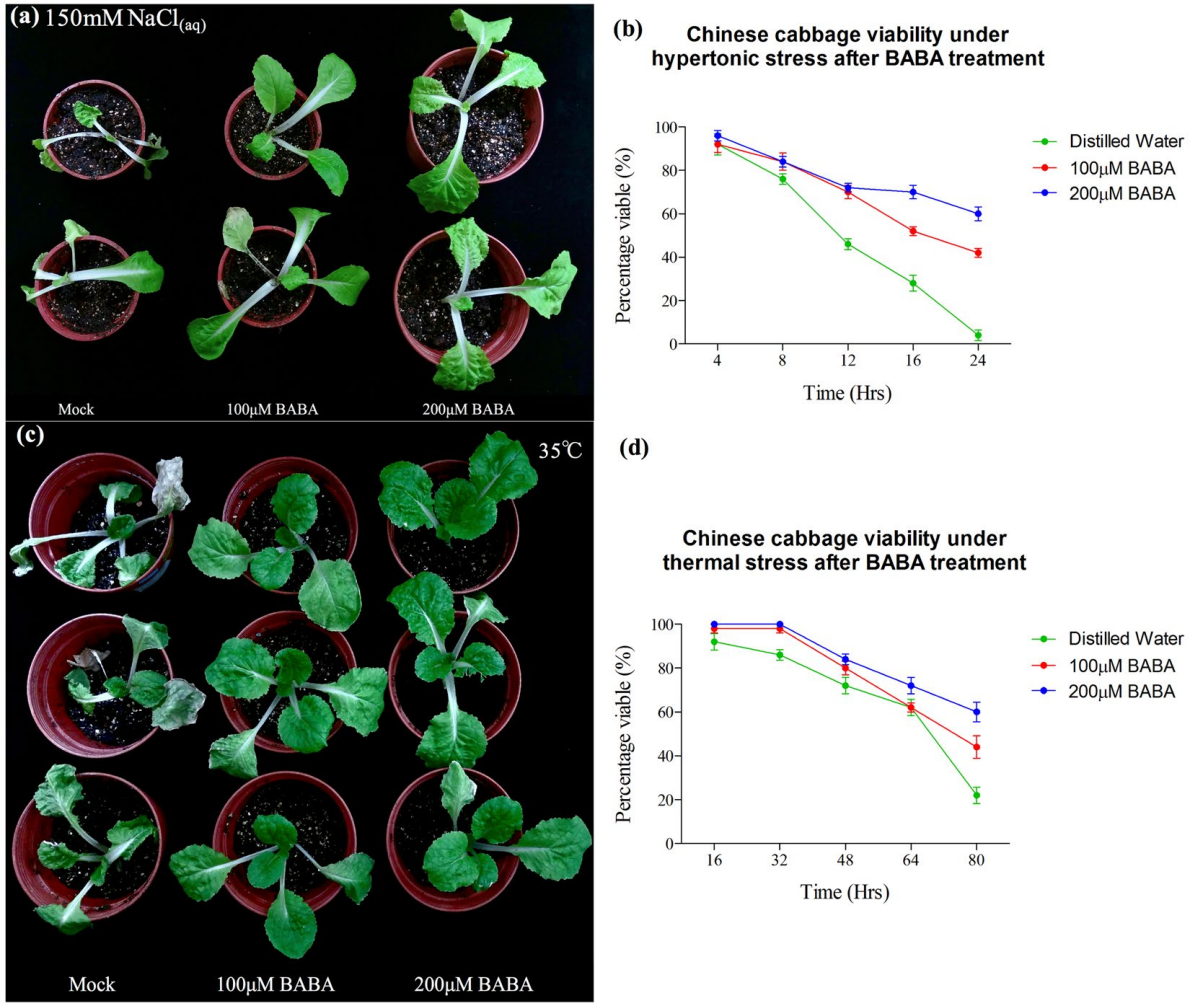
Exogenous application of β-aminobutyrate unto Chinese cabbage showed growth effect, salt and heat tolerance. (**a**) and (**b**) 21 DAG Chinese cabbage was shown to have more 24-hours hypertonic tolerance (150 mM NaCl) after exogenously treated with 100 μM and 200 μM BABA as compared to those treated with mock (distilled water). (**c**) and (**d**) 21 DAG Chinese cabbage was shown to have a higher thermal-stress tolerance (72 hours of 35 °C incubation) after exogenously treated with 100 μM and 200 μM BABA as compared to those treated with mock (distilled water). Quantifications of viable Chinese cabbage (**c**) and (**d**) were recorded from biological replicates of 10 Chinese cabbage and plotted against time (hours) with normal standard error.

**Figure 5:**
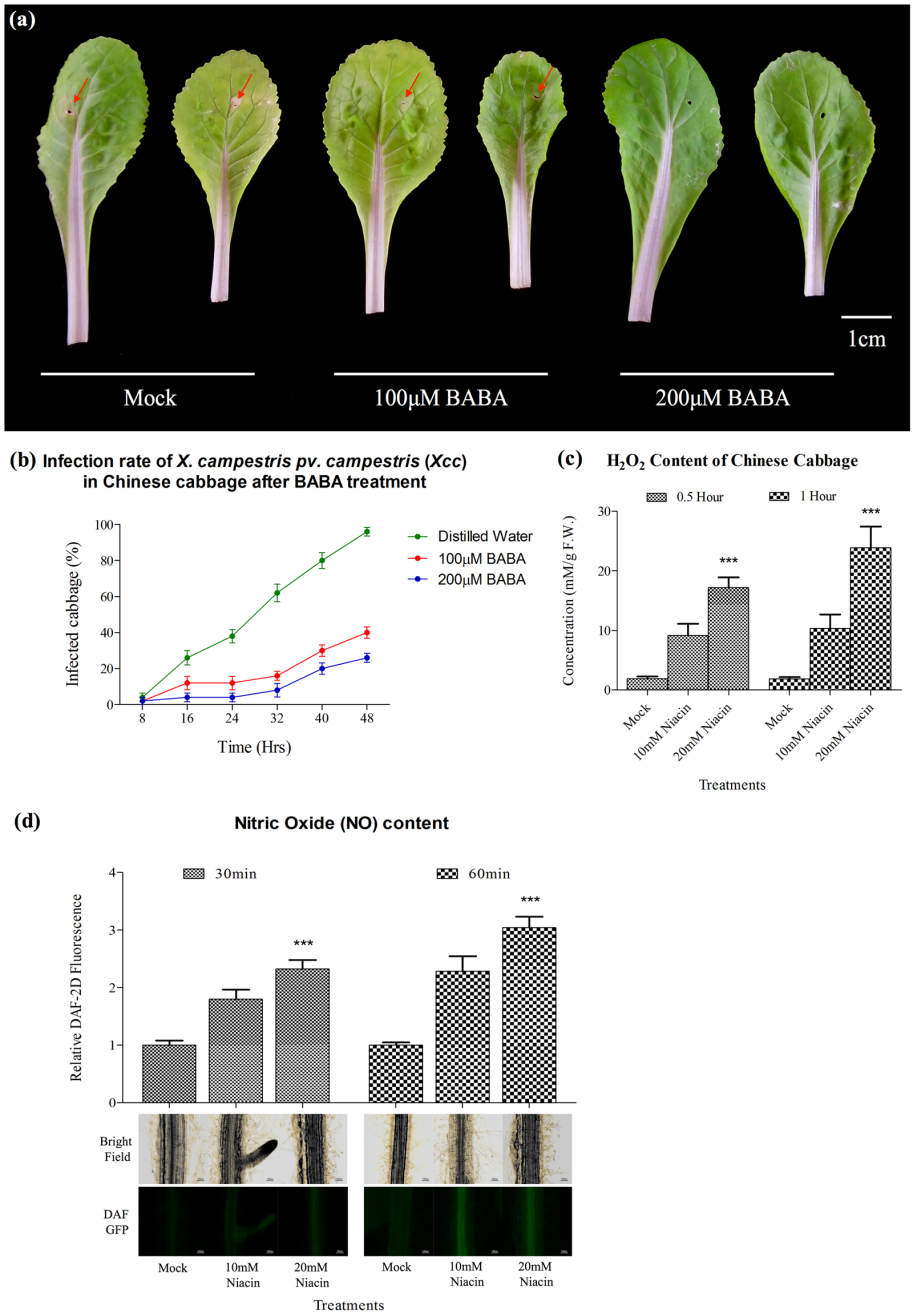
Exogenous application of β-aminobutyrate and niacin unto Chinese cabbage showed pathogenic tolerance and ROS/RNS generation. (**a**) and (**b**) 21 DAG Chinese cabbage were shown to have a higher immunity against *Xanthomonas campestris pv. campestris* (*Xcc*) (10^8^ c.f.u.) after treated with 100 μM and 200 μM BABA as compared to those treated with mock (distilled water). Red arrow denotes the necrosis symptom. (**c**) Chinese cabbages were shown to have higher ROS level in the form of H_2_O_2_ after half-hour and an hour exogenous treatment of both 10 mM and 20 mM niacin (**d**) Confocal-microscopic analysis showed Chinese cabbage treated exogenously with 10 mM and 20 mM niacin as compared to those treated with mock (distilled water) have a higher NO content in the root for both half-hour and an hour treatment. Quantifications of viable Chinese cabbage (**d**) were recorded from biological replicates of 10 Chinese cabbage and plotted against time (hours) with normal standard error. NO and H_2_O_2_ levels were measured from biological replicates of 6 Chinese cabbage and statistical analysis were performed with standard ANOVA comparing the marked column to mock treatment, where ns = non-significant; *p-value < 0.05; **p-value < 0.01; ***p-value < 0.001.

GC-MS analysis revealed an increase in GABA and a decreased in niacin contents during *P. indica* colonization (Figs 2b; 3d). To investigate a potential role of these two metabolites in the symbiotic interaction, exogenous application of GABA and niacin to Chinese cabbage was performed. As previously reported, BABA (β-aminobutyrate) functions similarly as GABA. It plays a priming compound to strengthen stress adaptation^28, 29^. Thus, we used BABA, instead of GABA, to apply exogenously on cabbage plants in this study. Twenty-four hours after spraying of a BABA solution (5 ml of a 100 μM or 200 μM solution) to the leaves, the seedlings displayed more tolerance to 150 mM NaCl and 72 h after spraying, their performance under high temperature stress (35 °C) was clearly better compared to the water-treated controls (Fig. 4a–d). Finally, if BABA-sprayed seedlings were inoculated with *Xanthomonas campestris pv. campestris* (*Xcc*), the black rot disease that affects *Brassicacae* worldwide, they performed better than the control seedlings (Fig. 5a,b).

Furthermore, treatment with niacin (10 mM or 20 mM) on Chinese cabbage seedlings for 0.5 or 1 hour caused higher H_2_O_2_ and NO production (Fig. 5c,d). These results are consistent with the idea that *P. indica* represses niacin and thus less H_2_O_2_ and NO production to allow more efficient root colonisation.

## Discussion

**Figure 6:**
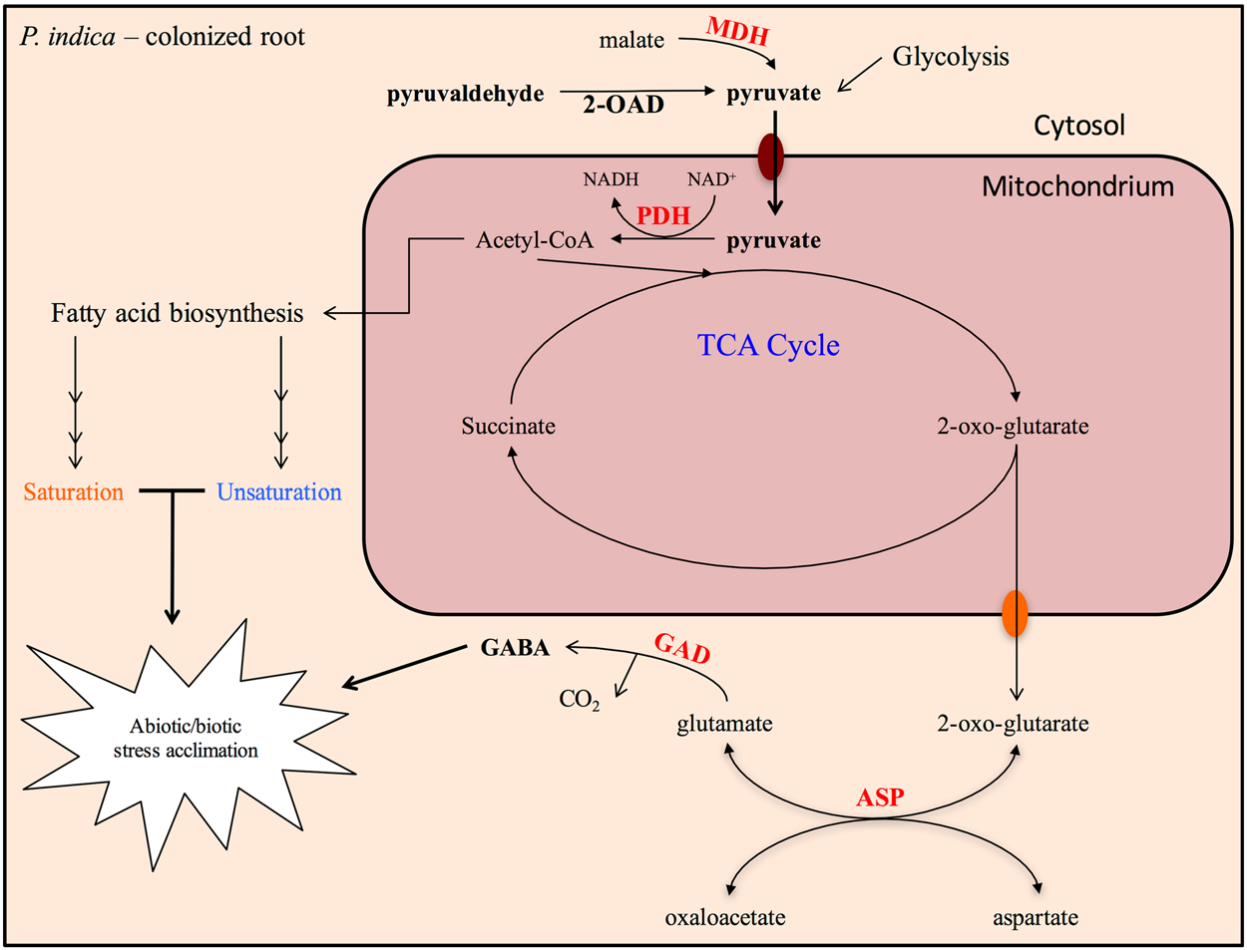
Proposed diagram showing the changes in metabolic pathways along with metabolites and genes induced by *P. indica* after colonization. Metabolites involved in the pyruvate metabolism are altered after 7 days of *P. indica* colonization in Chinese cabbage root. Elevation of metabolites involved in pyruvate metabolism consequently trigger (1) fatty acid biosynthesis and (2) TCA cycle, which produces 2-oxo-glutarate and elevates GABA. *P. indica-*induced GABA promotes abiotic stress acclimation and impedes growth of plants; whereas, L-glutamine or glutamate that could be produced via *P. indica-*induced pyruvate and –activated TCA cycle negates such effect as proposed^49^. List of genes induced by *P. indica* (marked in red) from previous EST-library^7^ ASP: aspartate aminotransferase. GAD: glutamate decarboxylase. MDH: malate dehydrogenase. 2-OAD: 2-oxaloacetic dehydrogenase. PDH: pyruvate dehydrogenase.

In this paper, we identified plant host metabolites, which are stimulated or repressed by *P. indica* to optimize root colonization and plant performance. As shown in Fig. 6, the pathways targeted by *P. indica* belong to the primary and secondary metabolisms, defense compounds, and signaling compounds. Their regulation by *P. indica* is consistent with the beneficial effect of the fungus on the performance of Chinese cabbage. In a previous study with root tissue of *A. thaliana*, Strehmel *et al*.^20^ demonstrated that nearly all metabolite levels increased upon co-cultivation in root extracts, except those in leaves. These included carbohydrates, organic acids, amino acids, glucosinolates, oligolignols, and flavonoids. Our current findings with Chinese cabbage resemble those from *A. thaliana*. Generally, the primary metabolism is activated, and the levels of metabolites in primary metabolism are increased. This indicates that *P. indica* can perform largely similar symbiotic function with Chinese cabbage and *A. thaliana*. Although some cases of different level, such as for pyruvate and niacin, were observed in Chinese cabbage when compared to *A. thaliana*. This might be caused by species-specific differences, but also by the fact that the response of Chinese cabbage to *P. indica* colonization seems to be stronger than the responses described for *A. thaliana*. Overall, the metabolomic profiles demonstrate that *P. indica* stimulates the energy status of the roots, trigger the accumulation of metabolites, which promote growth, and they are consistent with developmental processes and phenotypic changes.

We have demonstrated an elevation of metabolites involved in the pyruvate metabolism and detected higher levels of pyruvate, pyruvaldehyde and malate of the TCA cycle in colonized roots (Figs 2a and 6). Since pyruvate is located at the junction of assimilatory and dissimilatory reactions^30^, the higher the pyruvic and malic acid levels it could likely activate the host’s TCA cycle for energy generation required for root growth and consumption by the fungus. Furthermore, the fungus could provide additional pyruvate and malic acid to the roots via lactic and acetic acids, which accumulate during previously described fungal-growth in the maturation zone^7^. Pyruvaldehyde, which accumulates in *P. indica*-colonized roots, is a cytotoxic side-product of glycolysis and is synthesized from the pyruvate metabolism. It is detoxified to S-lactoylglutathione by lactoylglutathione lyase, which is induced for oxidative stress tolerance in plants^31, 32^. Moreover, lactoylglutathione lyase is identified in our previous EST library to be induced by *P. indica* after colonization of Chinese cabbage roots. Therefore, *P. indica* may stimulate the accumulation of these metabolites in this symbiosis to support the enormous growth requirement and stress adaptation.

Exogenous application of indoleacetamide (IAM) has led to the biosynthesis of auxin and the activation of auxin-responsive genes in *Oryza sativa*, *Sorghum bicolor*, *Medicago truncatula*, and *Populus trichocarpa* ^33^. The *P. indica* - derived IAM (Fig. 2d) that we observed is likely to stimulate auxin biosynthesis in Chinese cabbage roots. In the previous study, we reported that auxin and mRNA levels for auxin-signaling related genes were transiently increased during the early colonization period of Chinese cabbage roots^2, 7^. The increased level of auxin correlates with growth promotion and biomass production. Additionally, Hilbert *et al*.^34^ proposed that the IAM derived from *P. indica* plays an important role in the establishment of a mutual symbiosis with *Arabidopsis*, which may also be true for the interaction of *P. indica* with *B. campestris* roots. Also, Schafer *et al*.^13^ demonstrated a local accumulation of both plant and fungal IAA during the initial phase of the *P. indica*/barley interaction. These observations support our data that fungus-derived IAM stimulates IAA production and participates in promoting root growth. On the contrary, there were not any cytokinin and GA derivatives found in this study. These are in consistent with our previous studies in the subtractive EST library^2, 7^.

Aromatic amino acids, such as tryptophan, phenylalanine and tyrosine contribute to the high fluxes of fixed carbon through the shikimate pathway, and this can vary between 20 to 50%^35, 36^. We observed different regulation of aromatic acids and defense-related precursors: while some of them are up-regulated (e.g. phenylalanine, and 4-hydoxy cinnamic acid), others are down-regulated (in particular tryptophan). Within the plant kingdom, the phenylalanine metabolism along amino acid metabolisms are important for the synthesis of phenol derivatives such as coumarin, monolignal, lignin, spermidin, flavonoid and tannin. Genes involved in plant phenolic secondary metabolism such as phenylammonia-lyase^37^, which is frequently used as case studies of plant defense evolution^38^, has been identified in our EST library^2, 7^ (also in Supplementary Fig. S1). Similarly, important for a more efficient host-colonization, tryptophan, the precursor of many defense compounds or compounds with antimicrobial activities is decreased in *P. indica*-colonized cabbage roots (Fig. 2d). This suggests that tryptophan is intensively utilized to synthesize IAA, defense compounds, such as alkaloids, flavonoids, lignins and aromatic antibiotics^36^, which also lead to enhanced antimicrobial tolerance. To our knowledge, such regulation has not yet been reported in previous studies. Both metabolomic and transcriptomic analyses suggest that *P. indica* induces compounds from the phenylalanine metabolism for adaptation to prevail environmental conditions; while tryptophan is required for root defense and IAA-mediated root growth. This is consistent with the idea that the strongly growing roots in the symbiosis must be prepared for stress situations, e.g. by priming early stress responses through the mild induction of stress-related processes. On the other hand, the fungus must ensure that its nutritional state is balanced which is achieved by the sufficient amino acid supply from the host^39^.

GABA has been reported as a non-proteinous amino acid functioning as an important neurotransmitter in animal systems^21^. It has been found intra- as well as extracellularly, and proposed to be involved in the interaction of plants with a wide range of other organisms including pathogenic fungi, but also participates in developmental processes^22, 40, 41^. GABA is synthesized from glutamate by the root-specific glutamate decarboxylase^40^ (Fig. 6). The process is essential for sustaining ROS levels in plants^42^. Additionally, it has been shown that GABA production was prominent in the mitochondria in the presence of ROS to be metabolized along with pyruvate by GABA transaminase (GABA-T) to alanine and succinic semialdehyde (SSA)^43^. SSA is toxic for plants and there are two known pathways for SSA metabolisms: oxidization to succinate via SSA dehydrogenase (SSADH) or reduction to *γ*-hydroxybutyrate by glyoxylate reductase^44^. SSADH is located in mitochondria^45, 46^, and appears to be the main enzyme for SSA removal since mutants for this enzyme suggest its significant role in heat stress adaptation and development^22^.

BABA (β-aminobutyrate), a GABA homolog, was shown to induce resistance against root- and soil-borne pathogens in tomato and cotton, as GAGA^47, 48^. The perception mechanism of BABA has been identified in *Arabidopsis*. Recently, BABA was detected in *Arabidopsis* and several plants including *B. rapa*, *Z. mays, etc*.,. It is very clear that BABA was naturally present *in planta*, and having identical function with its isomers: AABA (α-aminobutyrate) and GABA (γ-aminobutyrate)^28, 29, 49^. In agreement with previous studies, our treatment of BABA confirmed that the homologous priming component, GABA induced by *P. indica*, could activate a PAMP-triggered immunity (PTI) response against pathogen infections (Fig. 5a,b).

Niacin or nicotinic acid is involved in the ROS burst in response to fungal infection. *A. thaliana* mutants *fin4-1* and *fin4-3* which lack *de novo* niacin biosynthesis, has lower ROS levels after fungal infection and does not triggered PTI or other immune responses^50^. Our results show that niacin-treated Chinese cabbage produces higher levels of nitric oxide and H_2_O_2_ (Fig. 5c,d). The lowered level of niacin occurring during symbiotic interaction implicates that it is downregulated to reduce plant immunity to facilitate the successful colonization of *P. indica* (Fig. 3d). Moreover, our analyses also show that the level of dimethylallylpyrophosphate (dimethylallyl-PP) is decreased (Fig. 3d). This metabolite is an intermediate for zeatin production and required for the mevalonate pathway to synthesize terpenoids, cytokinins and sterols^51, 52^. Even though terpenoids and cytokinins were not identified in the GC-MS analysis, the fungus is highly specific in regulating defense compounds in the host roots by lowering cholesterol derivatives which again may favor root colonization (Supplementary Fig. S2b). Despite lower ROS/RNS and sterol levels in colonized roots, the plants were not prone to pathogen (*Xcc*) attack (Fig. 5a,b). Previous findings showed that *P. indica* stimulated JAs and SA in *A. thaliana* for biotic stress adaptation^53^. Thus, it is conceivable that ROS production is only one defense compound among others and the sum of their regulation allows the fungus to balance defense and growth responses of the host. Moreover, earlier findings in *A. thaliana* showed that the amount of several cytokinins (isopentenyladenosine, *cis*-zeatin riboside and *trans-*zeatin riboside) were induced while others were not affected or decreased^54^. The reduced dimethylallyl-PP level detected in Chinese cabbage could also lead to lower cytokinin levels which ultimately establish a cytokinin-to-auxin ratio favorable for root growth.

Plants change their fatty acid composition to acclimate to higher temperatures: polyunsaturated fatty acids decrease and saturated fatty acids increase upon increasing temperature^55–57^. Consequently, mutants deficient in lipid unsaturation show enhanced thermal tolerance^24, 58, 59^. Oleic acid and arachidonic acid were shown to act as signaling molecules that modulate plant stress responses^60, 61^. We have observed that *P. indica* colonization increase the production of saturated fatty acids and decrease the production of unsaturated fatty acids in *B. campestris*. In our study, the increased level of polysaturated fatty acids and the decreased polyunsaturated fatty acids observed in *P. indica-*colonized roots suggested that the cell membrane fluidity is higher and innate immunity is lower. When plants are challenged by pathogens, lipase are activated to release unsaturated fatty acids which are subsequently transformed to oxylipins with diverse functions against microbial and insect attacks^25^. JA and its precursors, oxo-phytodienoic acids are regulating defense mechanism and balance growth and defense responses^26^. Although the oxylipin levels were not separately analysed in our work, the JA level is higher in *P. indica*-colonized roots (Supplementary Table 1), and the linolenic and arachidonic acid levels are reduced (Supplementary Table 2). These results demonstrate that *P. indica* colonization of Chinese cabbage roots may stimulate oxylipin biosynthesis via the degradation of unsaturated fatty acids.

Plants perform a tradeoff to optimize growth and acclimation to biotic and abiotic stress^62, 63^. *P. indica* promotes growth under drought stress in Chinese cabbage suggesting that the fungus shifts the balance towards growth^2, 7, 64^. Regulation of the levels of pyruvate, GABA, niacin and fatty acids by *P. indica* might be important to shift the balance towards growth promotion, in parallel enhancing the ability to respond to biotic and abiotic stress. Furthermore, pyruvate and L-glutamate as the intermediates of GABA biosynthesis are likely targets of the fungus to allow better plant performance under stress (Fig. 6). In conclusion, the identified metabolite changes induced by *P. indica* influence the biochemistry of the roots and might participate in the genetic reprogramming of root development, the symbiotic interaction, stress adaptations, and pathogen defenses. Finally, the interplay of auxin with other metabolic pathways need to be investigated in more details and at the cellular level, in particular since growth processes are controlled by local phytohormone maxima which are likely altered by *P. indica* in colonized roots.

## Materials and Methods

### Growth conditions of Chinese cabbage seedlings and *P. indica*, and estimation of plant growth promotion

Seeds of Chinese cabbage (*B. campestris* subsp. *chinensis*) were donated by Ming-Hong Seed Company, Taichung City, Taiwan. Seeds were sterilized with 75% alcohol for 10 min, then placed on a petri dish containing ½ MS nutrient medium^65^. Plates were incubated at 22 °C under continuous illumination (100 μmol m^−2^ s^−1^) for seed germination.

Seven days after seed plating on ½ MS medium, the growing seedlings were transferred to fresh plates containing ½ MS medium. One to six seedlings were used per petri dish and one fungal plague or one agar plague without fungus of 5 mm in diameter per seedling was placed at a distance of 1 cm from the roots. The colonized seedlings along with uncolonized ones were incubated at 22 °C under continuous illumination (50–55 μmol m^−2^ s^−1^). *P. indica* was cultured as described previously^66, 67^ on Kaefer medium. Seedlings were removed from the plates 7 days after co-cultivation and the roots were used for the extraction of secondary metabolites and GC-MS analyses. The results are presented on the basis of root fresh weights. Alternatively, the seedlings were transferred to pots and grown in a walk-in growth chamber, as described previously^11^.

### Secondary metabolite extraction and partitioning from plant and fungal material

All chemical reagents were of analytical grade. The silylation reagents were MTBSTFA [N-Methyl-N-(**t**-butyldimethylsilyl) trifluoroacetamide] and the extraction reagents were ethylacetate, acetone and distilled water.

Roots from seven-day-old Chinese cabbage (*B. campestris* subsp. *chinensis*) were harvested from 5 to 7 Petri dishes containing ½ MS nutrient medium (Murashige and Skoog 1962). The stems and leaves were disposed. Equal sample amounts of seedlings inoculated with *P. indica* or without *P. indica* were used for metabolite extraction. The three samples [roots with *P. indica* (colonized root), roots without *P. indica* (uncolonized root) and *P. indica* mycelium (hyphae)] were immediately frozen in liquid nitrogen after removed from the whole plant. The frozen samples were then incubated overnight (16 h) in a Labconco FreeZone® Freeze Dry System which was pre-chilled with liquid nitrogen.

The samples were removed from the freeze dryer, weighted and kept in liquid nitrogen under low light conditions [<100 μmol m^−2^ s^−1^]^68^ to prevent degradation of light-sensitive secondary metabolites. The three samples were then ground under the same light conditions with mortar and pestle prior to incubation in 100% ethanol overnight in the dark. The ethanol was removed with a Hydrion Scientific® rotary evaporator in the dark and the remaining material was weighted.

Partitioning of the secondary metabolites from the three samples was performed with ethyl acetate (EA) and water. 500 ml EA and 50 ml of milli-Q water were added to each sample in the dark. The suspensions were sonicated for 5 minutes before churning in order to dissolve the secondary metabolites in the heterogeneous mixture of EA and water. The two-phase suspensions of the three preparations were incubated overnight in the dark phases to obtain optimal phase separation, and the phases were then separated with separation funnels in the dark. The EA phases were used for further analyses and the solvent was removed in the dark with a rotary evaporator. The partitioning was carried out three times and the water fractions (containing mainly polysaccharides, proteins and unstable compounds) were discarded. Finally, the weights of the dried EA extracts were determined.

### GC-MS data analysis

The analysis was performed as described by Gobel and Feussner^69^. Dry materials of the three samples *Hyphae*, *Uncolonized root*, and *Colonized root* were re-suspended in GC-MS grade methanol (200 ng/μl). Silylation was performed using MTBSTFA under 80 °C for 2 h. The separation conditions on an Rtx-5MS column (30 m × 0.25 mm × 0.25 μm) for GC-MS analysis was set as follows: column flow, 1 ml/min helium; injector temperature, 250 °C; GC temperature program: 40 °C for 1 min, increase to 300 °C in 10 °C/min changes, 300 °C for 8 min; solvent delay, 470 sec; transfer line temperature, 280 °C; pegasus acquisition rate, 10 spectra/sec; mass range saved: m/z 50–600; ion source temperature: 200 °C. GC-MS was performed using the Pegasus^®^ 4D GCxGC-TOFMS from Leco.

Three sets of data were collected from three independent biological repetitions; and analyzed using the program ChromaTOF^®^ Software from Leco, by setting the threshold for up to 650 hits on m/z similarity and compared with the metabolites present in libraries of the software. Compounds with peak-areas below 700 were undetectable and compounds with less than 650 hits for m/z identification were removed. The identified compounds without silylation were individually analyzed; annotated and synthetic compounds were removed. Their abundance was calculated and quantified relatively to the internal control nonadecanoic acid-trimethylsilyl ester, which was added before the extraction and partitioning procedures and GC-MS analyses. Undetected peaks of any given compound from any given sample preparations: *Hyphae*, *Uncolonized Root*, or *Colonized Root* were calculated using threshold value of 700 for peak area. Moreover, the identified compounds were mapped to metabolomic pathways using the KEGG online software.

### Bioinformatics

The metabolites identified from the GC-MS were analyzed with the MetPA online software to position them into global metabolic pathways. The analytical settings for MetPA were used for *Fisher’s Exact* test. The MetPA software annotated compounds of *P. indica*-colonized and uncolonized roots, as well as those of *P. indica* hyphae were matched to *A. thaliana* and yeast databanks. Additionally, MetPA software contains a series of programs with matches for the metabolic pathways found in their databases, and assigns the compounds to these pathways with their *p-*values. Figures and tables were generated from the MetPA analysis.

### Exogenous application of β-aminobutyrate and niacin on Chinese cabbage for phenotypic and reactive oxygen species (ROS) analysis

One set of Chinese cabbage seeds were sterilized as previously mentioned above, each set containing eighteen, and then were sown unto petri dish containing 1/2 MS nutrient medium^65^. Five days after germination, they were transferred to ½ MS as well as ½ MS containing 100 μM and 200 μM filter-sterilized (45 μM filter) BABA. All six seedlings from one set and each treatment were recorded for stem and root length in centimeters, while two seedlings from each set of treatments were taken for phenotypic analysis by taking photograph of them aligned with each other.

Two more sets of Chinese cabbage seeds were sterilized, each set containing six, and then were sown unto petri dish containing ½ MS nutrient medium^65^. Five days after germination, they were transferred unto ½ MS as well as ½ MS containing 10 mM and 20 mM filter-sterilized (45 μM filter) niacin (Sigma-Aldrich, CAS 59–67–6, synonym to nicotinic acid); one set for 30 minutes and the other set for 1 hour respectively. Each set was used for NO quantification by following protocols using diaminofluorescein (DAF-2DA)^70^ staining and H_2_O_2_ by following procedures using titanium oxalate.

Twenty-seven Chinese cabbage seeds were sterilized as mentioned above and sown into twenty-seven 6-inch pots containing autoclaved soil/vermiculite mixture. After fourteen days, they were grown into Chinese cabbage plantlets and were used to perform thermal, high-salinity and biotic stress tolerance experiments, as described below in the following procedure.

### Exogenous application of β-aminobutyrate on Chinese cabbage for phenotypic analysis after thermal-stress and biotic-stress treatment

Two sets of Chinese cabbages, each set containing nine 21- DAG plants, were used for the thermal and biotic stress analysis. One set was subjected under a 72 hours thermal stress (35 °C) with three plants treated with 100 μM BABA and another three with 200 μM BABA by spraying each plant with 10 mL of respective concentration unto their leaves. The last three plants were sprayed with 10mL-distilled water, since the solvent vehicle for BABA was distilled water. After 24, 48, and 72 hours, nine of the plants were taken for phenotypic changes observation and a photograph was taken.

The second set of Chinese cabbages were used for a 24 hours high-salinity stress analysis. Exogenous application of BABA was identical to what was mentioned above. However, 150 mM of dissolved NaCl was used for the high salinity treatment for all nine plants. After 24 hours, phenotypic changes were recorded by taking photographs of the plants.

The final set of Chinese cabbages was used for a 72 hours biotic stress analysis. Exogenous application of BABA was identical to what was mentioned above. Here, *Xanthomonas campestris pv campestris* (*Xcc*) was used as the pathogen to induce infection in Chinese cabbage. The third leaf of the whole plant from each set was firstly pierced with a hypodermic needle. Next, they were infiltrated with O.D. 0.2 or 10^8^ c.f.u. amount of *Xcc* through the piercings. After 72 hours (which is the tested time for 0.2 O.D. amount of *Xcc* to infect Chinese cabbage with symptoms of rotting on leaf), the leaves from each treatment: water, 100 μM and 200 μM BABA were cut and photographed.

## Electronic supplementary material

Supplementary Information
