# Supplementary material for: Metabolomic compounds identified in Piriformospora indica-colonized Chinese cabbage roots delineate symbiotic functions of the interaction
Source: Sci Rep. 2017 Aug 24;7:9291. doi: 10.1038/s41598-017-08715-2 (PMC5571224; doi:10.1038/s41598-017-08715-2)
Supplement: Supplementary file 1 — Supplementary Information [file 41598_2017_8715_MOESM1_ESM.pdf]

Metabolomic compounds identified in *Piriformospora indica*-colonized Chinese cabbage roots delineate the symbiotic function of root endophytic fungus

Moda Sang Hua[1], Rajendran Senthil Kumar[1], Lie-Fen Shyur[2], Yuan-Bin Cheng[3], Zhihong Tian[4], Ralf Oelmüller[5], Kai-Wun Yeh[1*]**

Supplementary Information

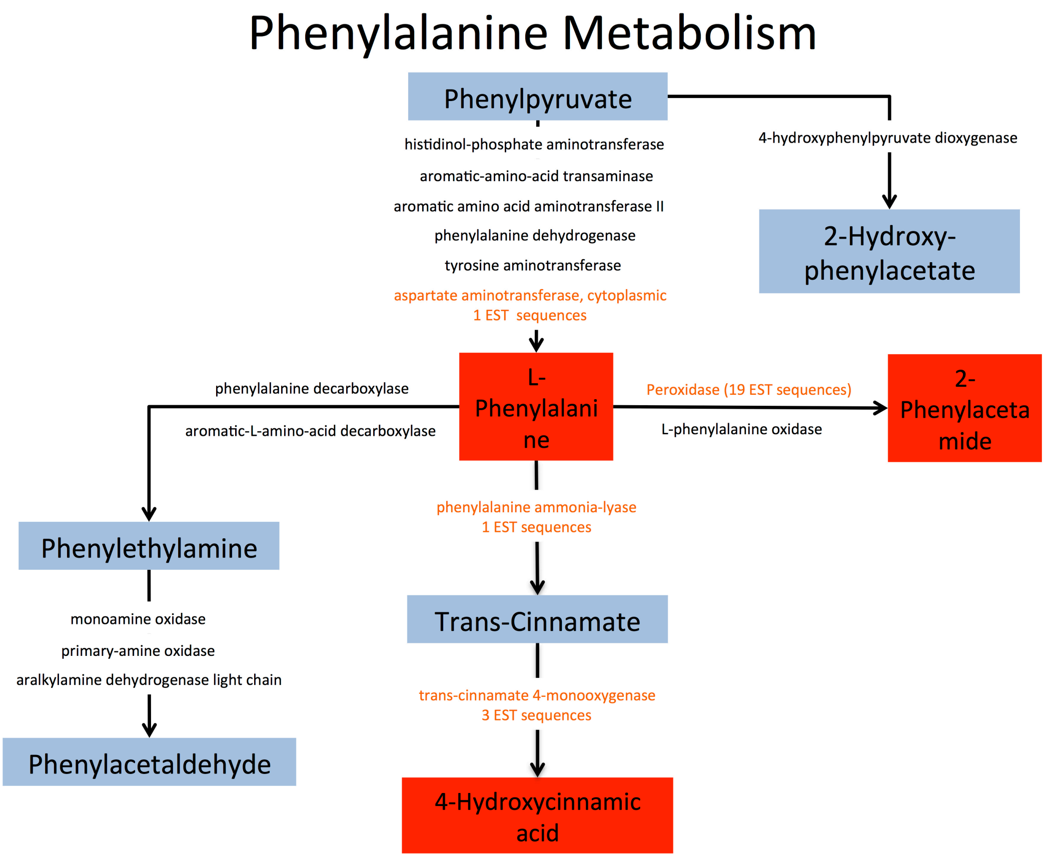

Supplementary Fig S1. Phenylalanine metabolic pathway along with genes induced by *P. indica* after colonization. Metabolites involve in Phenylalanine Metabolism such as L-phenylalanine, 2-phenylacetamide and 4-hydroxy cinnamic acid were shown to have increased after *P. indica* colonization. EST sequences mapped with subtractive EST analysis showed number of gene sequence found.

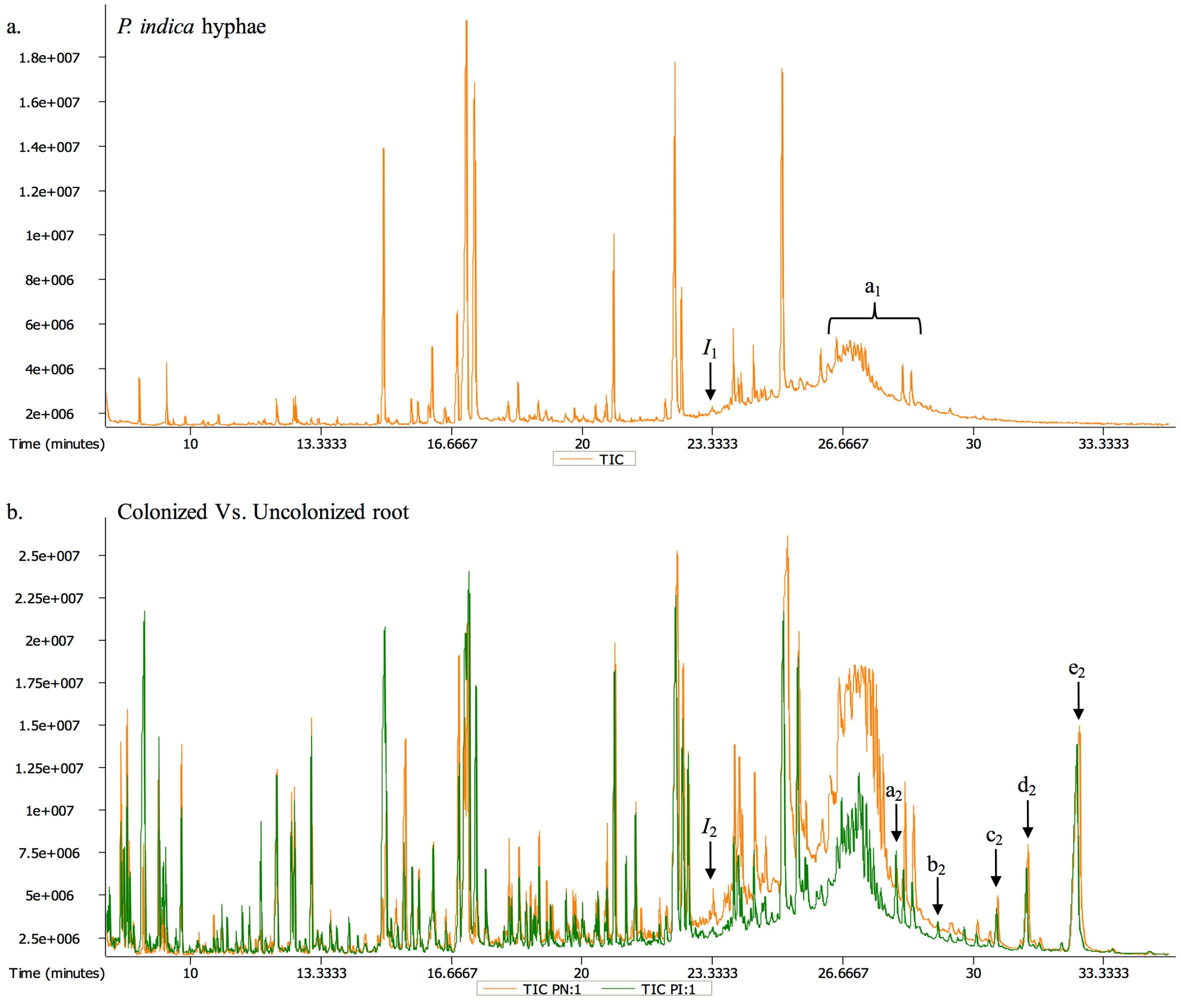

Supplementary Fig S2. Unknown compounds, cholesterol and terpenoid derivatives along with the internal standard (nonadecanoic acid-trimethylsilyl ester), shown in a chromatogram. (a) Chromatogram representing metabolites analyzed from 50µg/ml of sample prepared from *Hyphae* of *P. indica* where internal standard was loaded together (I_1 - internal standard [100ng]; a_1 – unknown compounds). (**b**) Merged chromatogram showing metabolites analyzed from 50µg/ml sample prepared from *P. indica*-colonized (green label) and uncolonized (orange label) root where internal standard was loaded together (I_2 - internal standard [100ng]; a_2 – terpenoid derivatives; b_2 – tocopherol; c_2 – cholesterol; d_2 – campesterol; e_2 – stimasterol). Metabolites annotated were taken from five replicates having similar peaks. Within each replicate, these annotated metabolites (except internal standard) have very low ionic-ms-ms hit points to be identified from the library.

Supplementary Tables

Supplementary Table 1. List of compounds identified by GC-MS specifically in: *Colonized root* and *Uncolonized root*. List of identified compounds generated by MetPA online software from the three libraries: *Hyphae*, *Uncolonized root*, and *Colonized Root* obtained by GC-MS analysis. Only compounds with peak area over 700 and appeared in all the three biological repeats were listed and compounds shared among *Hyphae* and *Colonized root*; *Hyphae* and *Uncolonized root*; and *Hyphae, Colonized root* and *Uncolonized root* were not included. *Fisher's exact test* was used for matching the compounds.

**Compounds specific in colonized root**	**Compounds specific in uncolonized root**
Agmatine	1,2-Dihydronaphthalene-1,2-diol
**(-)-Jasmonic acid***	1,2-Dihydroxy-8-carboxynaphthalene
(13E)-11a-Hydroxy-9,15-dioxoprost-13-enoic acid	2-Hydroxy-3-(4-hydroxyphenyl)propenoic acid
(S)-b-aminoisobutyric acid	2-n-Propyl-4-pentenoic acid
1-Aminocyclopropane-1-carboxylate	2-Pyrrolidinone
1-Benzyl-1,2,3,4-tetrahydroisoquinoline	4-Hydroxybenzoic acid
2-Phenylacetamide	4-Isopropenyl-2-oxy-cyclohexanecarboxyl-CoA
2-Phenylacetamide	5-Amino-2-oxopentanoic acid
2,3-Dihydroxy-3-methylbutanoate	Alpha-ketoisovaleric acid
3-Hydroxybutyric acid	Aniline
3-Indoleacetonitrile	**Arachidonic acid***
3-O-Sulfogalactosylceramide (d18:1/18:1(9Z))	Benzaldehyde
4-Hydroxycinnamic acid	Caffeic acid
8(R)-Hydroperoxylinoleic acid	Carnitine
Adipic acid	CE(10:0)
Aminoacetone	Diethanolamine
Arabinonic acid	Dimethylallylpyrophosphate
Benzoic acid	Formyl-5-hydroxykynurenamine
CE(22:0)	Geraniol
Chloroacetic acid	Gluconic acid
cis-1,3-Dichloropropene	Glycine

γ-aminobutyrate	Itaconyl-CoA
D-Galactose	Mannitol
D-Glucose	Myristoleic acid
D-Glucuronic acid	Phenanthracene
D-Ribose	Propionic acid
D-Xylose	Propyl alcohol
Diethylphosphate	Protocatechuic acid
Dodecanol	Tetracosanoic acid
Ethanol	Tetracosanoyl-CoA
Formaldehyde	Tetradecanedioic acid
Formamide	
Glycerol	
Hydrochloric acid	
Icosenoic acid	
Indole	
Indoleacetaldehyde	
Indoleacetic acid	
L-Phenylalanine	
L-Valine	
Maleic acid	
Malic acid	
Naphthalene	
Octadecanol	
p-Cymene	
Palmitic acid	
Phenylacetic acid	
Phthalic acid	
Prostaglandin A1	
Palmitic acid	

Pyruvaldehyde	
Pyruvic acid	
Salicin	
Terephthalic acid	
Tiglic acid	
Traumatic acid	
Tridecanoic acid	

Supplementary Table 2. List of compounds identified by GC-MS common among/between: *Hyphae*, *Colonized* and *Uncolonized roots* (24); *Uncolonized* and *Colonized roots* (72); *Hyphae* and *Colonized roots* (16); *Hyphae* and *Uncolonized roots* (11). Only compounds with peak area over 700 and appeared in all the three biological repeats were listed and compounds found specifically in *Colonized root* or *Uncolonized root* were not included. *Fisher's exact test* was used for matching the compounds.

Compounds common in: Pi Hyphae, uncolonized and colonized root	Compounds specific in uncolonized and colonized root	Compounds specific in Pi Hyphae and colonized root	Compounds specific in Pi Hyphae and uncolonized root
1-Phenanthrenecarboxylic acid	**γ-Linolenic acid***	Terephthalic acid	2-Furoic Acid
11-Amino[4.2.2]propella-2,4,7-triene	hexahydronaphthalene	1-Decene, 4-methyl-	4-Methyl-1,3-diaza-9,14-dioxaspiro[11.4]hexadeca-2,4-dien-8,10,13,15-tetraone
2,6-di-butyl-2,5-cyclohexadiene-1,4-dione	1,2,3,4-Tetrahydro-1,1,4,4,6-pentamethyl-5,7-dinitronaphthalene	1-Hydroxy-1-phenylpent-3-en-2-one	Cyclohexene, 4-(4-ethylcyclohexyl)-1-pentyl-
2-Ethylhexanoic acid	propen-1-naphthalene	5-Eicosene, (E)-	CE(10:0)
3-Butyn-1-ol	1-Iodo-2-methylundecane	9-Cyclohexylnonadecane	Diazene, dimethyl-
α-Linolenic acid	1-Isocyanatododecane	Benzenepropanoic acid, 3,5-bis(1,1-dimethylethyl)-4-hydroxy-, methyl ester	Octanamide, N,N-dibutyl-
Acetic acid	1-Oxa-spiro[4.5]deca-6,9-diene-2,8-dione, 7,9-di-tert-butyl-	Benzoic acid	Phenyl N,N-dibutylcarbamate

Azelaic acid	1-oxy-6'-methoxy-1',2',3',4'-tetrahydronaphthalenyl)-2-buten-1-one	Ethanone, 1,1'-(1,4-phenylene)bis-	Phthalic acid, bis(7-methyloctyl) ester
D-LACTIC ACID	10-Heneicosene (c,t)	Adipic acid	Propanoic acid
Dodecanoic acid	L-Tryptophan	Methyl 1,3-dihydro-2H-isobenzofuran-4-carboxylate	Propan-2-ol
Dehydroabietic Acid	1H-Pyrrole-2,5-dione	Phthalic acid, nonyl pentadecyl ester	Hydrogen sulfite
Formamide, N,N-dibutyl-	Arabinofuranose	3-Mercaptopyruvic acid	
L-Octanoylcarnitine	Tetramethyltricyclo[5.4.0.02,9]undecane	Dodecanedioic acid	
Oxalic acid	2-(PHENYL)PROPYL	Heneicosane	
Oleic acid	2-Aminobenzoxazole	Hexadecanedioic acid	
Pentadecane, 7-methyl-	Propenoate	Myristic acid	
Pentadecanoic acid	Pyroglutamic acid		
Sebacic acid	3,5-dimethoxy-benzaldehyde		
Squalene	Phytol		
Pelargonic acid	3-Ethyl-4-methyl-1H-pyrrole-2,5-dione		
Palmitoleic acid	7-METHYLHEPTADECANE		

LPA(16:0/0:0)	Acetamide, N,N-dibutyl-		
Stearic acid	Benzocaine		
Indoacetamide	Butyric acid		
	Bis(2-methoxyethyl) Phthalate		
	Campesterol		
	Cholesterol		
	Cinnamic acid		
	Cyclotetradecane		
	DINONYL PHTHALATE		
	Diisobutylphthalat		
	Diisononylphthalate		
	Arachidic acid		
	Ferulate		
	Caproic acid		
	2-Hydroxy-4-isopropenylcyclohexane-1-carboxyl-CoA		
	L-Proline		
	3-Dehydro-L-threonate		
	Linoleic acid		
	Mesityl oxide		
	Monoethyl malonic acid		
	N-OCTOXYETHANOLETHER		

	Nicotinic acid		
	OXYCARBONYL-2-PHENYL ETHENE		
	Suberic acid		
	Oxacyclotetradeca-4,11-diyne		
	Valeric acid		
	Phenyl dibutylcarbamate		
	Sinapate		
	Sucrose		
	L-Serine		
	Stigmasterol		
	Tert-Butyl isocyanide		
	Tri-m-cresyl phosphate		
	Undecylcyclohexane		
	[1,1'-Bicyclopropyl]-2-octanoic acid, 2'-hexyl-, methyl ester		
	Erucic acid		
	isophthalate		
	methyl palmitate		
	n-Octyl acetate		
	Sphinganine		
	sphinganine, 2-methyl-		
	trans-sinapinic acid		

	α-Tocopherol (vitamin E)		
	β-Sitosterol		
	2-Decenal		
	Chalcone		
	Succinic acid		
	Diethyl Phthalate		
	L-threonine		
	Phenylpropanolamine		
	Urea		
